# Healthy environments for athleTes (HEAT): environmental conditions along a 90 km ultra-marathon event, South Africa

**DOI:** 10.1007/s00484-024-02703-8

**Published:** 2024-06-13

**Authors:** H. Havenga, D. Gharbi, N. Sewry, B. Language, F. H. Neumann, J. M. Finch, T. Hill, J. Boulter, E. Jordaan, S. J. Piketh, M. Schwellnus, R. P. Burger

**Affiliations:** 1 Unit for Environmental Sciences and Management, North-West University, Potchefstroom, South Africa; 2 Sport, Exercise Medicine and Lifestyle Institute (SEMLI), Faculty of Health Sciences, University of Pretoria, Pretoria, South Africa; 3 International Olympic Committee Research Centre, Pretoria, South Africa; 4 School of Agricultural, Earth and Environmental Sciences, University of KwaZulu-Natal, Durban, South Africa; 5 Comrades Marathon Association (CMA), Medical Director, Pietermaritzburg, South Africa; 6 Biostatistics Unit, South African Medical Research Council, Cape Town, South Africa; 7 Statistics and Population Studies Department, University of the Western Cape, Cape Town, Western Cape South Africa

**Keywords:** Endurance events, Climate change, Heat stress, Aerospora, Environmental impacts on Athletes, Southern Africa

## Abstract

This paper provides an overview of the HEAT (Healthy Environments for AthleTes) project, which aims to understand the impact of environmental conditions on athlete health and performance during major sporting events such as long-distance running, cycling, and triathlons. In collaboration with the SAFER (Strategies to reduce Adverse medical events For the ExerciseR) initiative, the HEAT project carried out a field campaign at the 2022 Comrades Marathon in the KwaZulu-Natal province of South Africa. The measurement campaign deployed seven weather stations, seven PM_2.5_ monitors and one spore trap along the 90 km route to capture spatially representative measurements of complex micro-climates, allergenic aerospora, and particulate matter exposure. The results indicate that runners were exposed to moderate risk heat stress conditions. Novel findings from this initial campaign shows elevated and potentially harmful PM_2.5_ levels at spectator areas, possibly coinciding with small fire events around the race day festivities. Our findings show values PM_2.5_ levels over the WHO 24-h guidelines at all stations, while 2000 µg/m^3^ at two stations. However, the lack of an acute exposure standard means direct health impacts cannot be quantified in the context of a sport event. The HEAT project highlights important aspects of race day monitoring; regional scale climatology has an impact on the race day conditions, the microclimatic conditions (pollution and meteorology) are not necessarily captured by proximity instruments and direct environmental measurements are required to accurately capture conditions along the route.

**Supplementary Information:**

The online version contains supplementary material available at 10.1007/s00484-024-02703-8.

## Introduction

During endurance events, such as marathons and cycling tours, athletes often face extreme and varying environmental conditions that can impact their performance. Course topography, elevation, surface characteristics, and weather conditions can vary over small spatial–temporal scales during competitions. One major challenge in researching the impact of micro-environments on athlete health and performance is the lack of precision of the instrumentation used. Currently, studies typically use station data from weather services that are placed according to World Meteorological Organization (WMO) standards (Jarraud 2008), and usually a significant distance from the course. Subsequently, these do not capture the varying environmental conditions experienced by athletes as they compete over diverse terrains, through urban heat islands, and altitudinal gradients (Grundstein et al. 2022). To ensure safe participation for athletes, spectators and organizers, it is important to examine micro-climates during sporting events from a public health perspective. Studies such as Salata et al. (2017) indicate that even on a relatively small 27-hectare urban campus, micro-climate conditions can vary considerably. In addition, understanding the impact of adverse environmental conditions, such as heat stress, biological pollutants (e.g. allergenic pollens and fungal spores), and air pollution, on physical activity and health is becoming increasingly relevant, as the frequency of heatwaves and extreme heat days may be rising due to climate change (Dahl et al. 2019; Liu et al. 2017; Tuholske et al. 2021; Raymond et al. 2020).

To understand the impact of changing environmental conditions related to heat, heat stress indices (de Freitas & Grigorieva 2015) have been developed to account for factors such as temperature, solar radiation, wind speed, clothing insulation, activity level (including levels for sporting participation), and its effect on human physiology. Although these indices are not specifically developed for sporting events, they have been used in various instances to assess performance of athletes and the incidence of medical encounters at sporting events (Thorsson et al. 2021; Gasparetto and Nesseler 2020; Hodgson et al. 2022). Guidelines from World Athletics, the International Institute for Race Medicine, and American College of Sports Medicine (Armstrong et al. 2007) utilize the Wet Bulb Globe Temperature (WBGT) during sporting events. Other indices that have been used in a sporting context include the Physiologically Equivalent Temperature (PET) (Thorsson et al. 2021), and the Universal Thermal Comfort Index (UTCI) (Honjo et al. 2018; Hollander et al. 2021, Konefat et al. 2021), or direct meteorological measurements without deriving a heat stess index (Knechtle et al. 2021). While heat stress indices are often limited in their representation of specific physical activities (Grundstein & Vanos. 2021), they do offer insight into the possible range of effects that can be expected under specific conditions. Dee et al. (2022) found that adverse environmental conditions for athletes, particularly exposure to increasing frequency of heatwaves, is projected under future climate change scenarios. These projections highlight the need for better understanding of extreme heat events for organizers and participants. The 2013 event was described as one of the warmest days the race has been held on in its history, only 55% of starters finished the race on this day (Comrades Marathon Association 2024), showing the potential effect of extreme heat days on this ultra marathon event.

Exposure to high levels of air pollution can have detrimental effects on athletic performance, particularly during endurance events (Rundell 2012). The particulate matter component of air pollution has been linked to decreased lung function, increased risk of heart disease, declining exercise endurance and a rise in airway inflammation (Pope et al. 2004; Rajagopalan et al. 2018), which could potentially impair athletic performance. Asthma and related allergy symptoms, such as wheezing and chest tightness, are reported by athletes after competing in endurance events such as cycling, swimming, or long-distance running (Helenius et al. 1998). The prevalence of allergic rhinitis in elite athletes has been reported between 15 and 29%, in contrast to wheezing, which is reported by 6% to 15% of athletes (Helbling et al. 1990; Helenius et al 1997; Helenius and Haahtela 2000; Maiolo et al. 2003; Nystad et al. 2000). Helenius et al (1997) found that the prevalence of physician diagnosed asthma and allergy symptoms in trained athletes was higher (17%) than that of a control group (3%), possibly due to increased exposure to pollen during spring and summer training and the inhalation of cold air during winter training.

Studies from global cities reveal that aeroallergens significantly negatively impact runners' respiratory health (Robson-Ansley et al. 2012). During the 2000 Sydney Olympics, Gioulekas et al. (2003) identified that 51% of athletes were atopic, and 16% had evidence of seasonal allergic rhinitis and/or conjunctivitis through questionnaires, physical examination and skin-prick testing. Allergic rhinitis (Alaranta et al. 2005) and allergic rhinoconjunctivitis (Katelaris et al. 2003, 2006) are more prevalent in athletes, often not diagnosed and undertreated. An aerobiological monitoring network was established at the Athens 2004 Olympic Games (Derman et al. 2010) to provide information on circulating aeroallergens in three Olympic cities, ensuring safety for allergic athletes who visited Greece from January to September 2004. The most frequently diagnosed allergenic pollen types, including Cupressus spp. (Cypress), *Platanus* spp. (Plane tree), *Olea* spp. (Olive tree), Poaceae (Grasses), and *Artemisia* sp. (Mugwort), and fungal spores such as *Alternaria* spp. and *Cladosporium* spp. were identified (Bonini et al. 2006). Given the situation, athletes should be adequately prepared before and during important sporting events for exposure to high allergenic pollen or fungal spore levels, and the environmental conditions should be monitored (Robson-Ansley et al. 2012). These findings from other scientific studies emphasize the importance of monitoring air pollution levels during athletic events and minimizing exposure to harmful pollutants. The Athens monitoring campaign (Derman et al. 2010) was an innovative and integrated monitoring approach that included a real-time sampling of bioaerosols to monitor pollen and/or fungal spores and meteorological parameters such as solar radiation, relative humidity, and temperature. The data was used in real-time to increase athletes' and organizers' awareness during the games. In a recent study, respiratory disease (asthma, allergic rhinitis) was the most common chronic disease reported by race entrants (2.4%) during the Comrades Marathon race (2014–2019) (Brill et al. 2021). To date, no studies have been conducted in South Africa to monitor the spatial and temporal variability of environmental conditions linked to a mass participatory sporting event. This presents an opportunity to improve the current knowledge regarding environmental conditions locally.

Considering the limitations in our understanding of the environmental conditions during sporting events mentioned above, improved planning and preparedness for outdoor activities is necessary, particularly in light of changing environmental conditions. Effective forecasting, pre-screening, and race day monitoring can help to increase awareness of the possible effects of warm or polluted environments on athletes and to provide preparation recommendations for organizers and participants (Heathcote et al. 2018; Périard et al. 2015). Here we present an overview of the first study at the Comrades Marathon to quantify environmental conditions at high spatial resolution. The project provides data for organizers, policy makers and researchers to better understand the micro-climates at specific events.

## Data and methods

### Study area

**Fig. 1:**
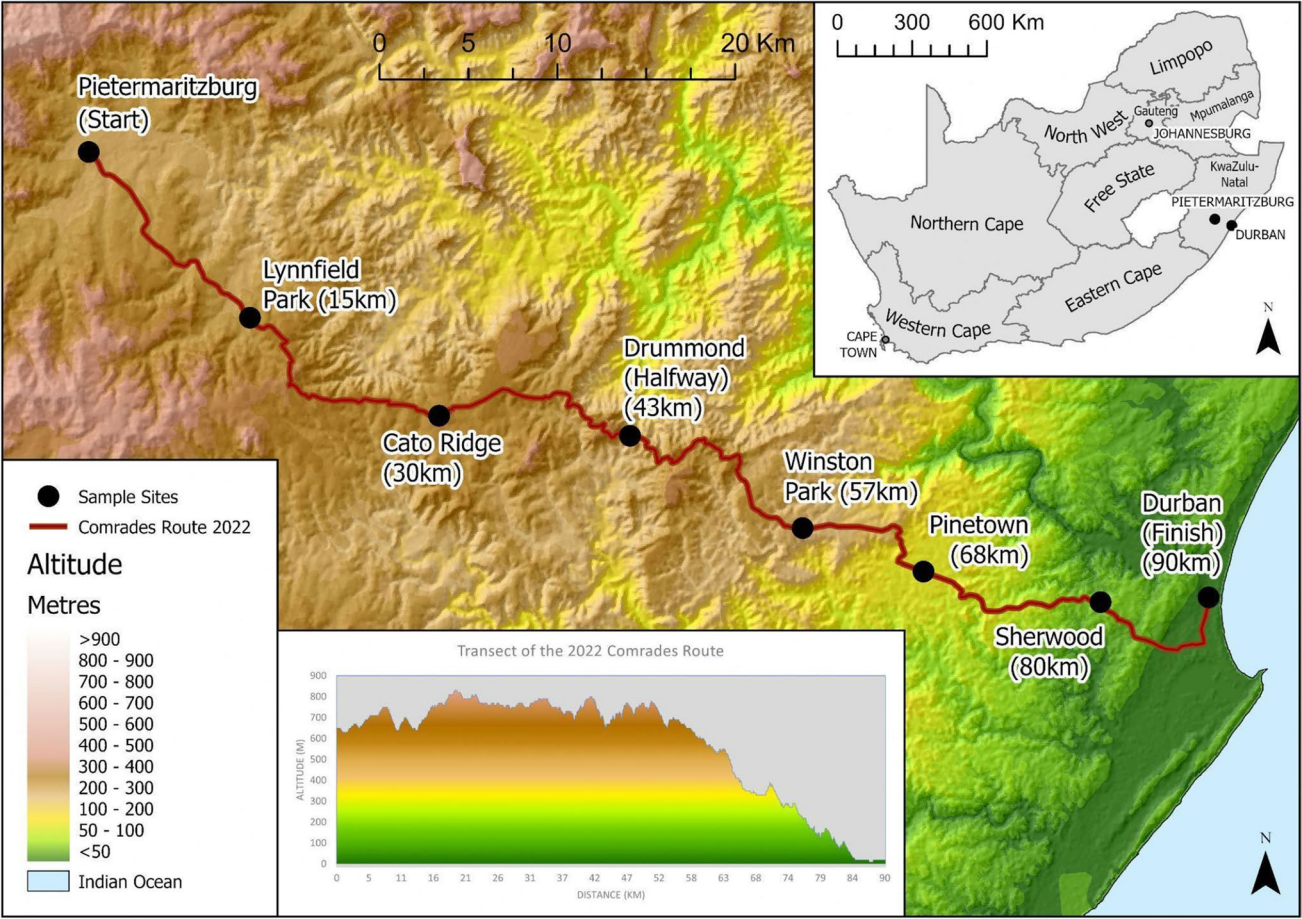
Map of the deployment points along the route, each point is + -15 km apart. At each cut-off point, a station was located. In total 7 stations were deployed, with the first deployment at 15 km (Lynnfield Park)

The Comrades Marathon is an annual ultra-marathon event held in South Africa and is recognized as the largest road ultra-marathon in the world, attracting approximately 15,000 participants per annum. The 90 km long route stretches from Pietermaritzburg to Durban, with the starting city alternating each year (Fig. 1). In 2022, the event was a “down” run, with runners starting in Pietermaritzburg and ending in Durban. The route presents a unique and challenging topography resulting in challenges related to weather parameters since different climatic zones are transected. Durban (6–8 m a.s.l.; − 29.86°; 31.02°), one of the starting locations, is a coastal city with a Cfa (Temperate without a dry season and hot summer region) climate, while Pietermaritzburg (654 m a.s.l.; − 29.60°; 30.38°) lies on the transition between the Cfa and Cfb (Temperate with Dry Winter and Warm Summer) zones. Along the coast of the Indian Ocean, the region falls within the subtropical Indian Ocean Coastal Belt Biome (IOCB) (Mucina et al. 2006). The IOCB is characterized by a mosaic of subtropical grasslands, forest patches with diverse tree species (e.g., on dunes), wetlands, coastal lakes with mangroves and swamp forests, and has cultivated land with sugarcane and pine plantations and subsistence farming (Mucina et al. 2006). The area between Durban and Pietermaritzburg consists of a series of hills, valleys and urban / peri-urban areas that athletes must traverse. The popularity of the event, distance covered by athletes, and high incidence of medical encounters, all contributed to the initiation of the first HEAT field deployment in 2022.

**Fig. 2:**
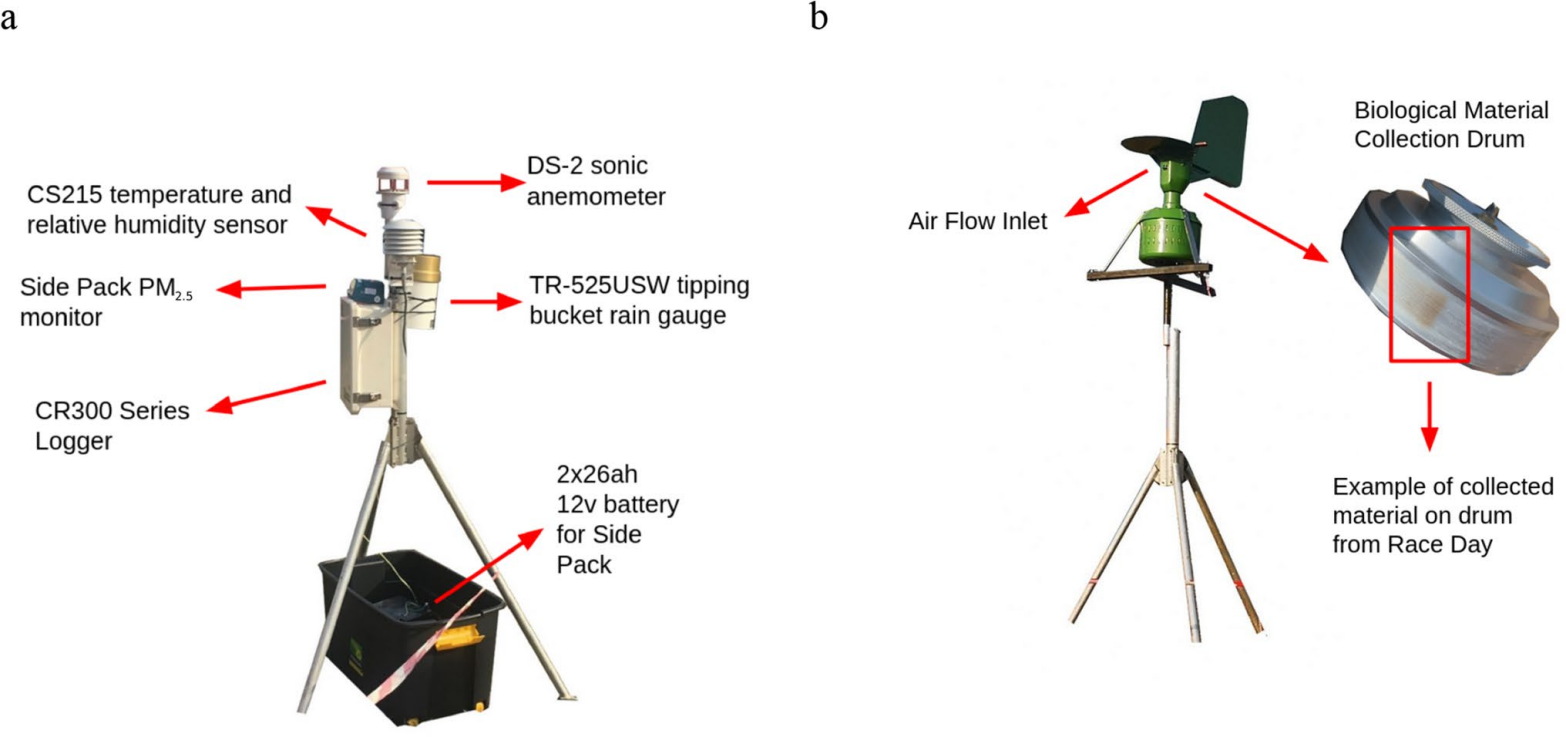
Instruments placed along the route. **a** The meteorological station and the PM_2.5_ SidePak Monitor. **b** Burkard® spore trap, which was placed at Halfway point

The trial field deployment was initiated during the 2022 event and was based on two considerations. First, from a health and physiological perspective, the exact conditions runners experienced along the route were monitored at each cut-off point. These are locations along the route where runners who have not passed by a certain time are prevented from continuing. Instruments and weather stations were placed at each cut-off point (approx. 15 km apart), which will allow for future field campaigns to correlate the time, pace, and position of each athlete with the specific environmental data. The first record at each station starts when the first runner passes the station, and ends when the cut-off time stops runners at the point from running further. In Table 1 the location of all the stations on the route is described with the corresponding distance and instruments placed on the route. At each site, stations were located in direct proximity to the route. Second, the station and researchers' mobility along the route was considered. Due to security concerns, the instruments were deployed on the morning of the event. To accommodate this, the station was designed to be mobile, with a small footprint, and could be deployed and taken down within 5 to 10 min. Each station (Fig. 2a) consisted of a DS-2 sonic anemometer, CS215 Campbell Scientific temperature and relative humidity sensor, TR-525USW tipping bucket rain gauges, and a SidePak PM_2.5_ measurement device. A Burkard 7-day volumetric spore trap (Fig. 2b) to measure allergenic pollen, spores and fragments was set up at the Halfway point of the race. The Halfway point was selected for the placement of the spore trap due to availability of continuous (120v) electricity to power the instrument. The meteorological stations were powered by a 7ah 12v battery, while the SidePak was powered by a 2 × 26ah 12v battery. AC power was provided at the Halfway point for the spore trap. Both the weather station and PM^2.5^ data was collected at 1 min intervals at all stations. At Lynnfield Park (15 km) a SidePak malfunctioned and at Winston Park (57 km) battery error led to data loss (see Table 1). SidePak Flow was calibrated at site to 1.7 l/min, and all instruments were zero calibrated before deployment.

**Table 1:** Data Retrieval for the 2022 Field Campaign

Location	Instruments Status	Time deployed *(Total time; station turned on (first runner passing); station turned off; cut-off for last runner)*
Start—0 km	Data gathered from the University of KwaZulu-Natal weather station (see UKZN 2022)	15 min; 05:30—05:45
Lynnfield Park (Weather Station & SidePak)—15 km	100% data retrieval and SidePak 50% data retrieval, instrument malfunction	2 h 2 min; 06:21—08:23; Cut-off 8:10
Cato Ridge (Weather Station & SidePak)—30 km	100% data retrieval	3 h 2 min; 07:01—10:03; Cut-off 10:00
Halfway (Drummond) (spore trap, Weather Station & SidePak)—45 km	100% data retrieval	4 h and 48 min; 07:57—11:45; Cut-off 11:40
Winston Park (Weather Station & SidePak)—57 km	Instrumentation error	NA
Pinetown (Weather Station & SidePak)—68 km	100% data retrieval	5 h 25 min; 09:46—15:11; Cut-off 15:00
Sherwood (45th Cutting) (Weather Station & SidePak)—80 km	100% data retrieval	6 h 21 min; 10:26—16:47; Cut-off 16:40
Finish line (Weather Station & SidePak)—90 km	100% data retrieval	11:01—17:30 (6 h 31 min)

### Heat stress calculation

We used the Liljegren method to calculate Wet-Bulb Globe Temperature (WBGT) (Liljegren et al. 2008; Kong & Huber 2022) from the R Heatstress package (Casanueva 2019; Gerrett et al. 2019, Brimicombe et al. 2023) at all 7 stations. Solar radiation was estimated using the average radiation on the race day (08:00 am to 05:00 pm) from the UKZN station in Pietermaritzburg, 307W/m^2^. The calculation of WBGT would be improved with the use of radiation sensors at each station, which was not currently available during this study. This method does not account for other factors that can affect heat stress, such as clothing, activity level, and heat acclimatization, which should be considered when assessing the risk of heat stress. The WBGT heat stress risk level, as per the American College of Sports Medicine, was used in the study to categorize heat stress levels from no risk (“continue normal activity”) to high risk (“cancel event”) (Armstrong et al. 2007).

### Aerospora monitoring and botanical survey

Aerobiological monitoring was conducted at the Drummond Halfway point [45 km] (669 m a.s.l.; − 29.75°; 30.70°), using a standard Hirst-type seven-day recording volumetric spore trap (Burkard® spore trap) to collect outdoor air samples (aerospora; pollen and fungal spores). Sampling was performed on race day, Sunday, August 28th, following the South African Pollen Network (SAPNET) protocol (http://www.pollencount.co.za). The sampling area was selected based on the surrounding vegetation and the absence of physical barriers that may obstruct airflow around the spore trap. The aerospora sampling started at 2:00 am and continued for 10 h. The sampler operates on the principle of impaction through suction, with a 2 mm clockwise movement on the sampling drum over each hour, allowing the calculation of hourly airborne particle concentrations. The trap has a pumping mechanism that maintains a constant airflow of 10 L per minute, checked with a flow meter. Melinex® tape coated with a thin film of Vaseline® (petroleum jelly) was fixed on the rotating drum of the sampler to trap airborne particles. The drum was collected at 12:00 pm, and the tape was cut into fragments of 48 mm length and mounted on slides using glycerine jelly. Concentrations of pollen grains and fungal spores were expressed as pollen grains (p.g/m^3^) or fungal spores (sp.g/m^3^) per m^3^ of air and determined and counted along three horizontal transects with an Olympus light microscope at × 400 magnification. Hourly counts of airborne pollen and fungal spores were carried out with the aid of a grid attached to the back of the slide.

A botanical field survey of the sampling area was conducted the day after the marathon (August 29, 2022). The spore trap location was identified by GPS and the field survey was conducted along four 100 m transects aligned to the compass points North, South, West, and East (Figure S1). The aim of the survey was to obtain an understanding of the most likely local parent plant taxa contributing to the airborne pollen and spores, with special attention to flowering specimens. The transect along the western compass point was curtailed by private property and fencing. The area surveyed included a railway line, a major road, a parking lot, flower beds, mowed lawn area, a shopping complex, and private gardens. During the survey, each plant encountered was identified to the highest possible taxonomic level, and photographs or voucher specimens were taken where identification was challenging.

## Results

The following section details the results from instrumentation deployed along the event route. The results are intended to highlight the total environment that needs to be taken into consideration when examining the impact of environmental conditions (anthropogenic and natural) when doing field campaigns such as this. When the results were compiled we examined the synoptic environment to understand the large scale circulation patterns, which drives the subsequent surface weather and possible recirculation of atmospheric pollutants over the region (Tyson et al 1996), the direct measurements on the ground provides evidence of the microclimatic conditions. These microclimate measurements, covering large areas of the route, provide unprecedented insight into the possible environmental conditions of this event. While the novelty of the general campaign has been highlighted, it is worth noting that the deployment of the Sporetrap and the PM_2.5_ provide new insights not seen before in events of a similar nature. Following the results, the final section explores the findings with insights into the values reported and the possible implications.

## Synoptic Conditions

**Fig. 3:**
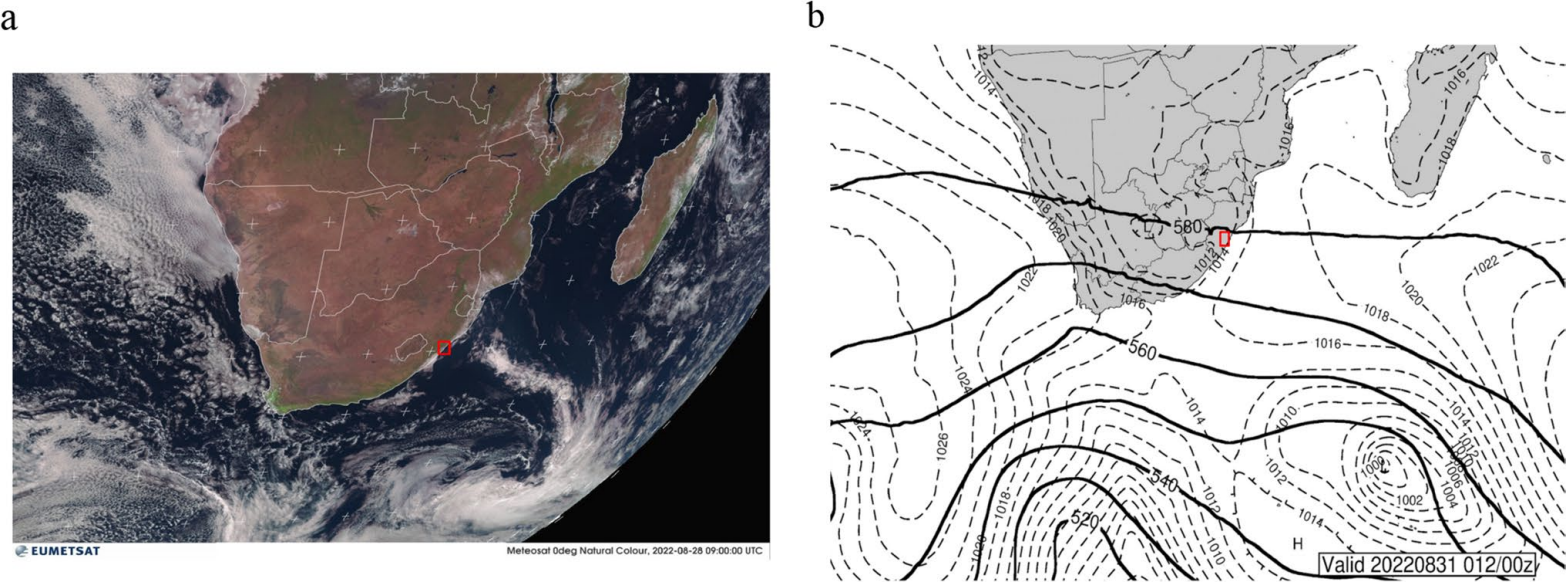
**a** Satellite imagery (©EUMETSAT 2022) of race day indicates mainly fine and dry conditions along the interior. The cold front south east of South Africa results in onshore flow along the escarpment and subsequent cloud formation. **b** Synoptic weather for 28 August indicates a weak pressure gradient between the passing cold front South East of the country and the easterly low over the interior. There was some onshore flow with light cloud development along the Durban coast. Westerly flow at 500 hPa is pronounced and would enhance cloud formation throughout the day along much of South Africa and along the race location (in both images indicated in red)

On August 28, the synoptic conditions over the study area were dominated by the passage of a cold front that lay to the south east of the country (Fig. 3a). Ridging anticyclonic air behind the front led to onshore flow over the eastern Escarpment, causing cloud formation along the east coast between Durban and Beira, Mozambique. These conditions led to cool to mild conditions along the race route by late afternoon. The synoptic map (Fig. 3b) indicates an easterly low visible across Northwestern South Africa, which would enhance surface moisture availability. In addition, the map indicates a strong westerly flow at 500 hPa across South Africa, which would favor cloud formation over the region. The large-scale circulation system provided favorable conditions for athletes to compete. The specific weather conditions along the route are presented in more detail below.

## Temperature and Relative Humidity

**Fig. 4:**
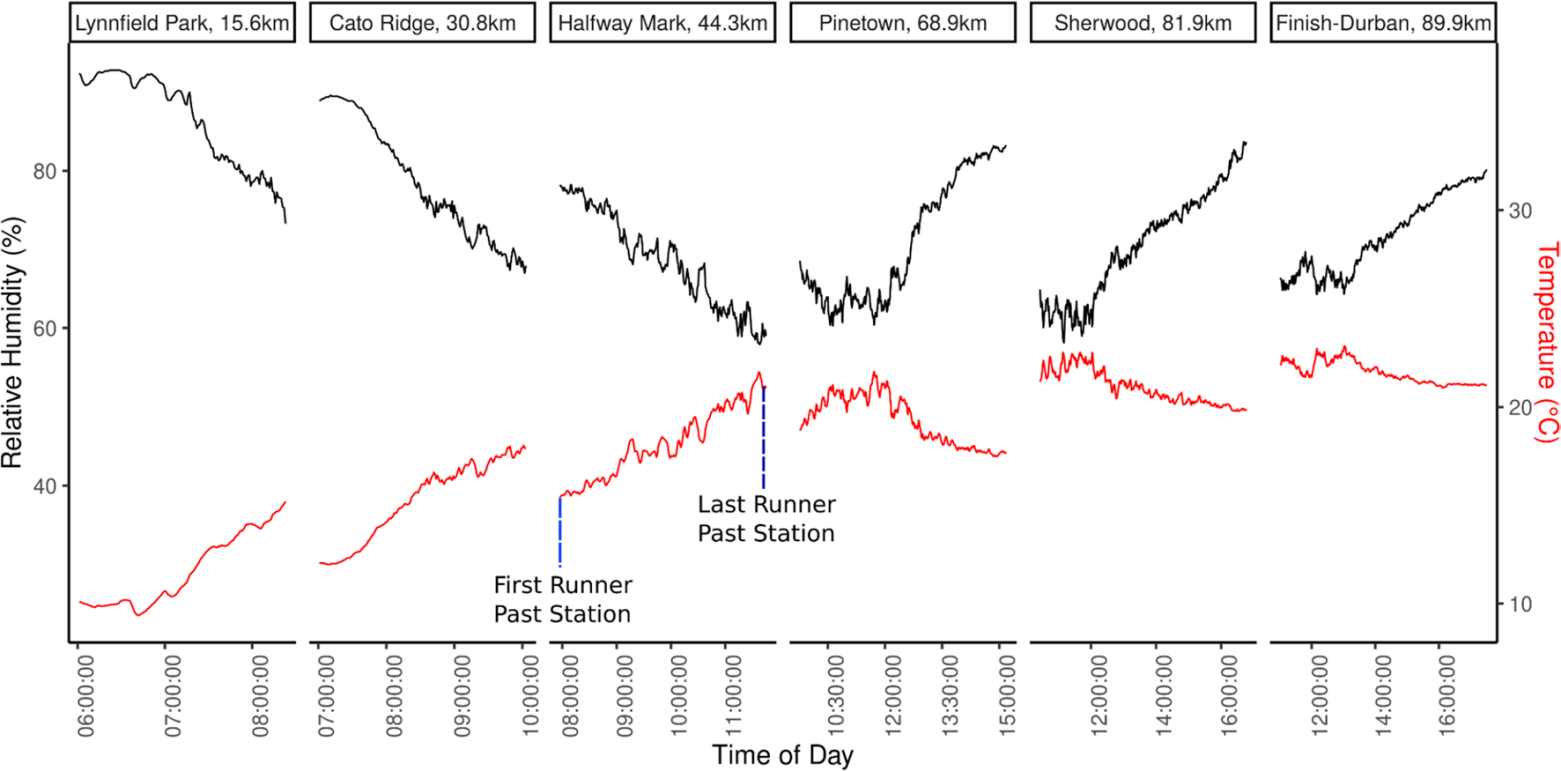
Temperature and Relative Humidity at all stations along the route. The first record at each station starts when the first runner passes the station, and ends when the cut-off time stops runners at the point from running further. The total deployment time of each station is documented in Table 1

Lynnfield Park recorded a minimum temperature of 10℃ at 06:42 am on race day, while the finish point registered the highest temperature of 23℃ at 13:02 pm (Fig. 4). Two factors contributed to this observation. First, the natural diurnal cycle of temperature influenced the increase in temperature, and second, the runners traveled from Pietermaritzburg to Durban, which is indicative of the change in altitude and the prevailing weather changed during the course of the day.

The recorded relative humidity ranged from 58 to 93%, with a median of 74% (25th-75th percentile: 67%-93%), reflecting conditions from relatively dry to very humid. Lynnfield Park, Cato Ridge, Drummond (Halfway point) and Pinetown (inland) had RH values ranging from 67–91%, with Lynnfield park having the lowest minimum RH of 73% and the highest maximum RH of 91%. This can be expected as the station at Lynnfield Park was the first station that runners passed and the first to be taken down as RH decreased. RH is often close to 100% in the mornings as the air temperature cools overnight, the air becomes saturated with moisture leading to a high level of humidity. Sherwood and Durban (coastal) had RH values ranging from 58–80%. However, the first runners only passed these stations in the afternoon, when the temperature usually rises due to solar heating, resulting in a decrease in relative humidity. There is also a sharp increase in RH around noon at Pinetown, Sherwood and Durban and this corresponds to a change in wind direction resulting in the advection of colder air from the southeast—with sea breeze characteristics. This would also increase RH as there is an influx of moist air and colder conditions affecting RH. There is an increase in RH later in the day close to sunset, as the air temperature again decreases and dew point temperatures are reached, leading to higher RH levels. In the following sections it is important to consider the relative humidity, temperature, wind speed and direction to understand the effect on the athletes. These variables, in combination, can indicate the amount of heat related stress faced by a runner.

### Wind Speed and Direction

**Fig. 5:**
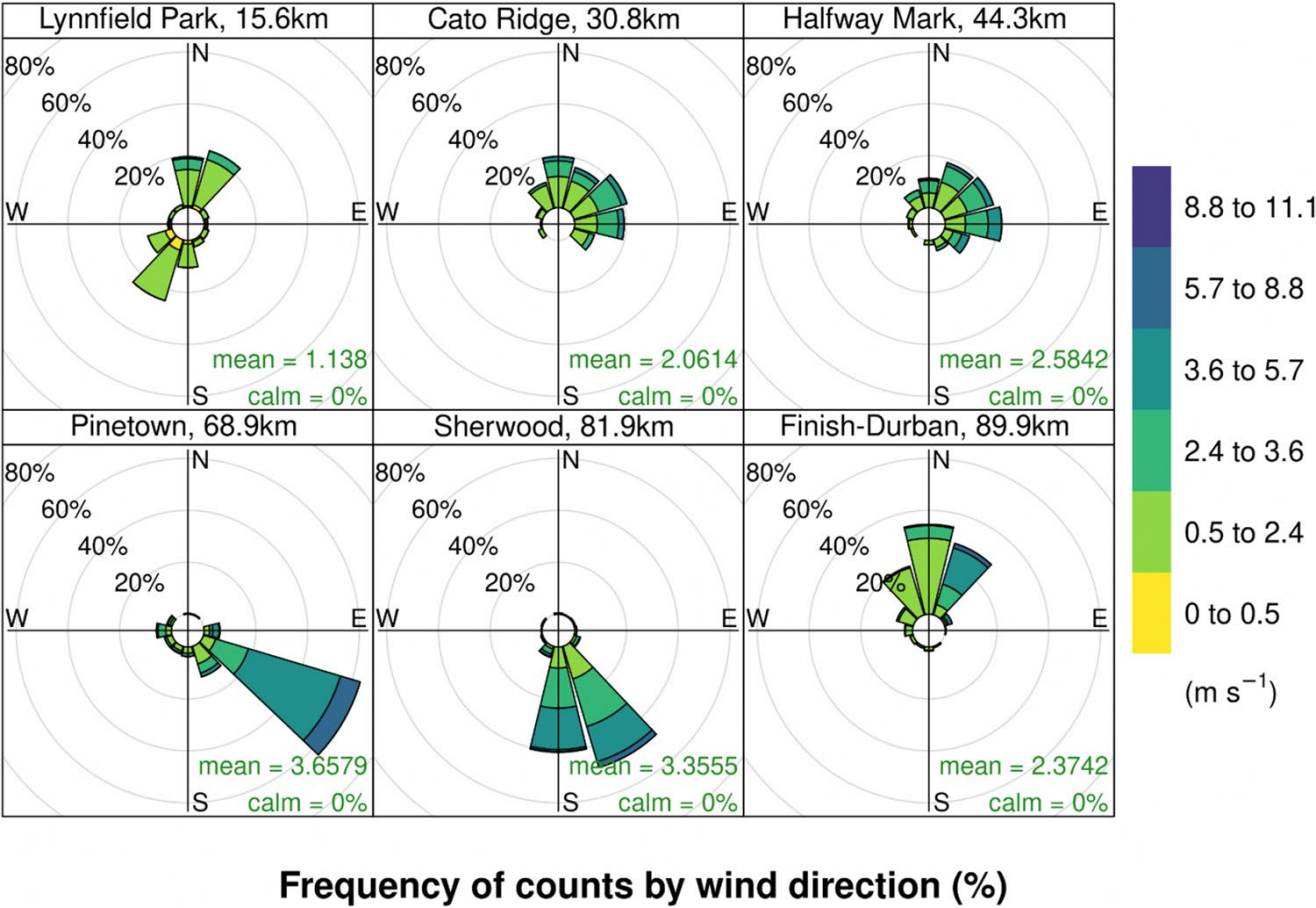
Frequency distribution of wind speed and direction at each station for the time period that runners passed the station. Data was recorded at 1 min intervals, each station data was averaged over the period that the station was deployed, i.e. from the first runner that passed, until the cut-off was reached as detailed in Table 1. Measurements show the local, microclimate winds at each location and are subject to various influences, but represent the conditions runners typically experienced

Wind conditions (Fig. 5) varied significantly between each station along the route. At Lynnfield Park (15.6 km), a NNE wind and SSW wind were observed. The site was situated next to the road with a small embankment, along which the wind would follow. Cato Ridge (30.8 km) and Halfway point (44.3 km) had similar wind characteristics, with most of the winds originating from the NE direction. This wind direction corresponded with the expected winds from the synoptic map around the dominant low pressure system observed on the day. The last three stations had variable winds, all were located in urban areas surrounded by buildings and trees, each with characteristic local effects. The dominant wind direction at Pinetown (68.9 km) and Sherwood (81.9 km) was predominantly SE and S, respectively, indicating that the wind was mostly moving parallel to the road. At the finish (90 km) the large stadium where runners finished would also change the wind. The final station was located at the stadium as the runners entered the stadium, the station was no more than 500 m from the finish area. The main reason for this placement is to have access to the station and to ensure no interference with participants and spectators at the finish area where large groups typically gather. The wind speed of 2-3 m/s represented a gentle breeze. The most notable impact of the wind speed was seen at Pinetown, Sherwood and the finish when at 12:00 a shift in wind direction resulted in a direct change in the temperature and relative humidity at these stations. At this time a south easterly wind resulted in a major change of the PM_2.5_ levels. This is discussed in the subsequent sections further.

### Calculated Wet-Bulb Globe Temperature (WBGT)

**Fig. 6:**
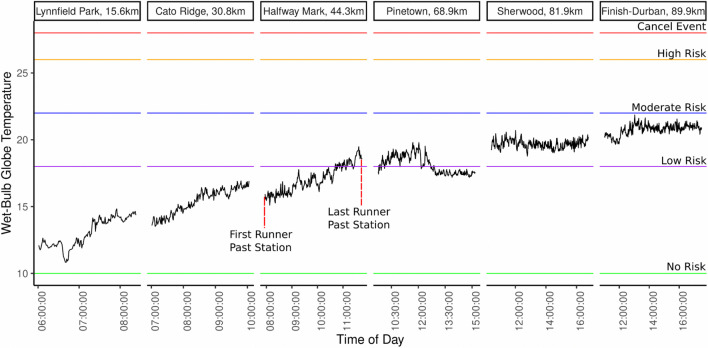
Derived wet-bulb globe temperature (WBGT) using Liljegren method. Low risk conditions occurred later in the day, runners could potentially be exposed to these conditions for 7 h

We report on the WBGT in this section. The WBGT (Fig. 6) takes into account not only temperature, but also humidity, wind speed, and solar radiation, making it a comprehensive indicator of heat stress. Other heatstress indices exist, but the use of the WBGT is intended as a reference to show the response that an average human might have under different conditions. Other indices, such as the Universal Thermal Comfort Index (UTCI), offers new insights into heat stress exposure based on dynamic clothing and advanced metabolic models (Psikuta et al 2012; Havenith et al 2012), future research into the applicability of different indices to endurance events can offer expanded insight into unique physiological response in endurance athletes. The American College of Sports Medicine (ACSM) recommends specific guidelines for managing activity in different WBGT ranges (Armstrong et al. 2007). During the event day athletes faced “no” *(normal activity)* to “low risk” *(Normal activity. Monitor fluid intake)* of heat-related issues throughout the event. “No risk” conditions were dominant while “low risk” conditions were only present during the afternoon (from 11:00 am to 6:00 pm) stages of the event. At Pinetown a slight decrease in the local temperature resulted in the lowering of the ACSM risk value. The drop in temperature at Pinetown corresponds with a change of wind direction and speed, throughout the early measurements there were calm conditions prevailing, at noon a south easterly wind developed advecting colder air from the nearby (+ -23 km) Indian ocean. This measurement is confirmed by a local South African Air Quality Information System (SAAQIS) station in New Germany, approximately 3 km northwest of the deployed site. The increase in wind speed also led to a decrease in PM_2.5_ levels at this station (discussed in the next section). This example also highlights the role of larger circulation in the local environment. In different regions of South Africa “berg wind” conditions dynamic with adiabatic heating of air results in significant temperature changes within short periods of time, often leading to health impacts (Heunis et al. 1995). The “low risk” conditions still warrant extra care by organizers, the results indicate that a runner could be exposed to these conditions for 7 h on this day if an athlete takes the full allowed time to compete.

### Particulate Matter

There are several considerations when examining the PM_2.5_ results we reported on. The SidePak monitor only measures the size fraction concentration, the composition and source of PM_2.5_ can vary significantly (Thangavel et al 2022). Our monitors and technicians at each station took several photos and reported that high PM values corrsponded to spectator activity. However, as we can see from the Pinetown values (Fig. 6) and the corresponding PM_2.5_ values at the same station around noon (12:00 SAST), local meteorology plays a major role (the instruments operate on different loggers, and power sources). During the mentioned time period a decrease in temperature resulted in a marked change in PM_2.5_ concentration, this could indicate a source that was not local had an impact on the PM levels and with a change in wind direction there was a change in the microclimate and the PM concentration. However, we mention this to highlight that understanding the source of PM_2.5_ could help determine sources and possibly reduce them on race day. PM_2.5_ sources can be of natural and anthropogenic nature and this needs to be examined further.

**Fig. 7:**
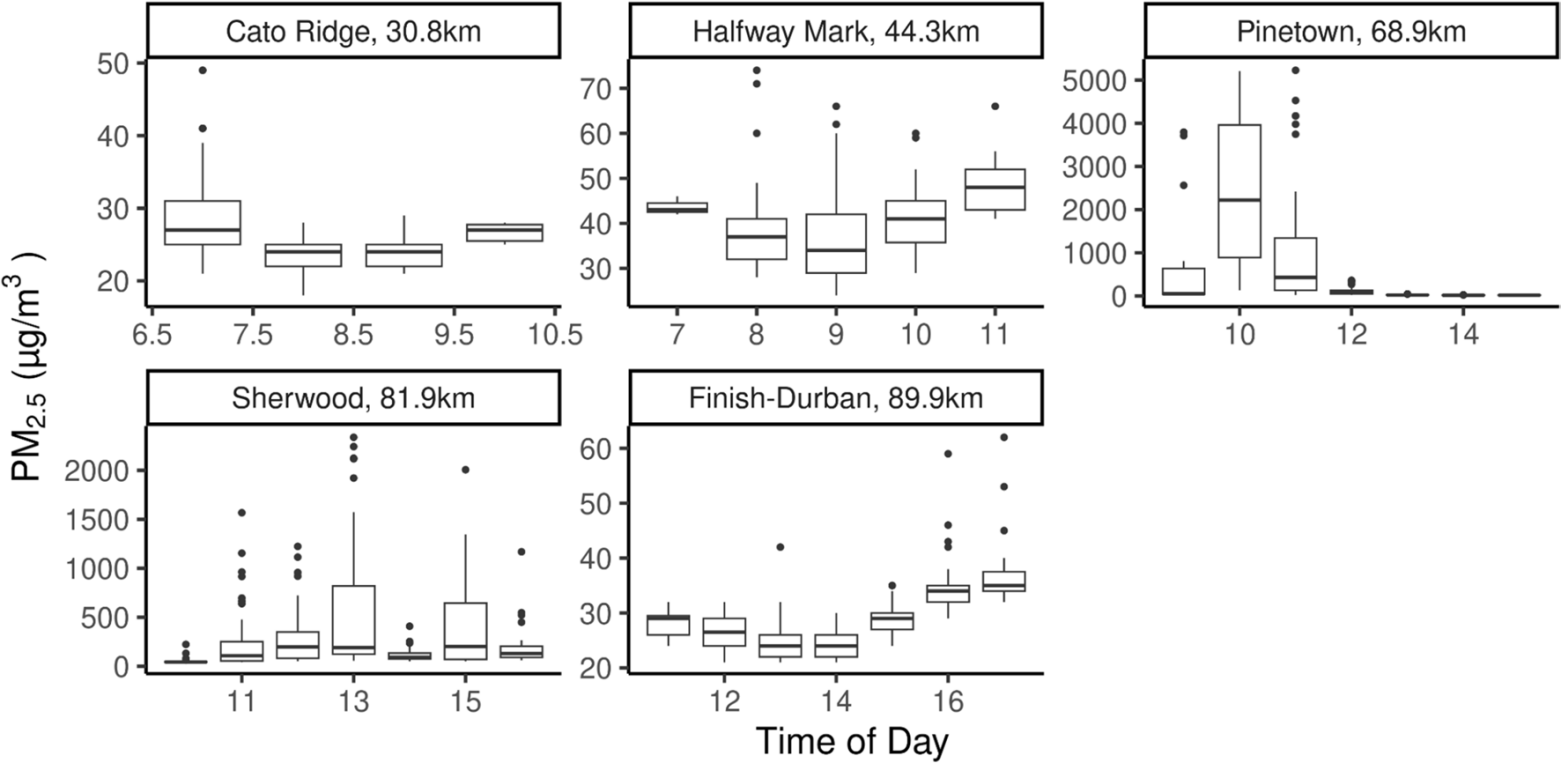
PM_2.5_ Concentration at all sites per hour is shown in the box and whisker which indicates the median, 25th and 75th percentiles of the raw data, including the outliers. A sudden drop in PM_2.5_ at Pinetown at noon corresponded to a change in wind direction, also observed at an nearby SAAQIS air quality monitoring station

For PM_2.5_, all data above the 99th percentile of the day's recorded PM_2.5_ were excluded. We applied this quality control to attempt remove extreme values and ensure data is not skewed towards them, the 99% values, could also be anomalies or unrepresentative values (although we do not believe this to necessarily be the case), removing these values ensures that single values that might be introduced by other factors is removed. In the case of our extreme values as we see in several stations, the removal of these values does not alter the median value recorded, but could skew the mean. After data above the 99th a maximum instantaneous value of 5227 µg/m^3^ at Pinetown was the highest value recorded. Concentrations over 5000 µg/m^3^ were noted three times at Pinetown (Fig. 7). The SAAQIS station at New Germany, located northwest of the station deployed next to the road, measured a maximum PM_2.5_ concentration of 17 µg/m^3^ at a similar time to our maximum measurement. Several notes have to be made here, the SAAQIS station is located in a suburban area, while the event route passes right in the middle of the central business district. Our high PM measurements correspond directly with our technicians observing bbq fires right next to the station by spectators, we did not intervene or attempt to move spectators away from the station as this was the exact conditions runners were exposed to in their immediate environment. The significant difference between the SAAQIS station and the microclimate supports our argument that proximity stations do not serve as a good representation of the environment athletes are exposed to and without direct measurements such as this we would not have seen the elevated PM_2.5_ levels. However, the local meteorology has a significant role to play, a turn in wind direction immediately led to the dispersion of pollutants at the Pinetown station.

At Sherwood, maximum instantaneous PM_2.5_ concentrations reached 2337 µg/m^3^. The site is located in an urban area where traffic was allowed in the opposite direction, unlike other sites with no public traffic in close proximity. This likely contributed to the median concentration of 126 µg/m^3^ at Sherwood, the highest among all sites. Cato Ridge had the lowest PM_2.5_ values, with a median of 25 µg/m^3^. Possible reasons include the early morning passage of most runners and a lack of spectators, and the absence of major smoke or traffic sources. The Halfway point recorded a median concentration of 46 µg/m^3^. This study revealed variations in PM_2.5_ concentrations across different locations, reflecting factors such as spectator behaviour (i.e. open fire for cooking), and traffic conditions on environmental conditions.

### Allergenic Agents

**Fig. 8:**
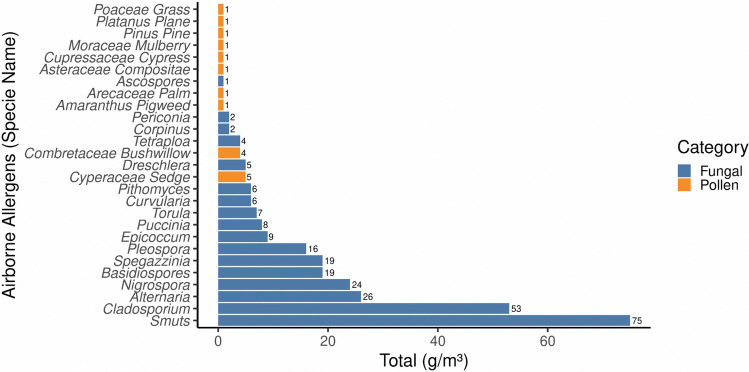
Fungal spores and pollen count at the Halfway point. Most abundant fungal spores were Smuts (75 sp.g/m^3^), *Cladosporium* (53 sp.g/m^3^), *Alternaria* (26 sp.g/m^3^). Cyperaceae (5p.g/m^3^). Combretaceae (4p.g/m^3^) were the abundant pollen

During the sampling period, a total of 303 aeroallergens per m^3^ were recorded, with fungal spores contributing the most at 284 sp.g/m^3^ (94%) compared to pollens at 17p.g/m^3^ (6%) (Fig. 8). The atmosphere contained 17 fungal spore types and 10 pollen types, with the most abundant fungal spores being smuts at 75 sp.g/m^3^ (26%), *Cladosporium* at 53 sp.g/m^3^ (19%), *Alternaria* at 26 sp.g/m^3^ (9%), *Nigrospora* at 24 sp.g/m^3^ (8%), and basidiospores at 19 sp.g/m^3^ (7%). The most abundant pollen types were Cyperaceae and Combretaceae, with 5 p.g/m3 and 4 p.g/m^3^, respectively, and the remaining types contributing 1 p.g/m^3^ (Fig. 8).

**Fig. 9:**
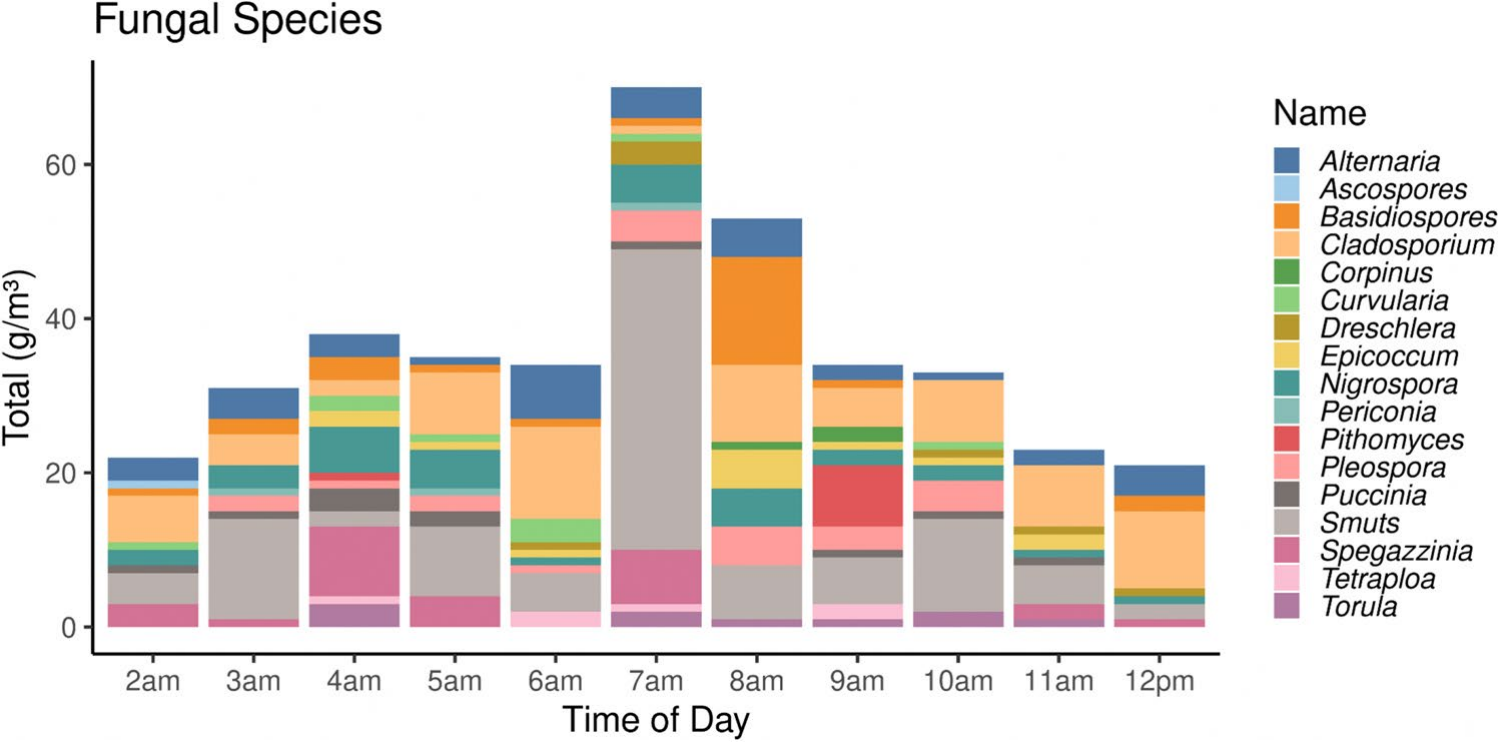
Fungal spores diurnally distribution pattern of airborne fungal spores (2:00am-12:00 pm)

**Fig. 10:**
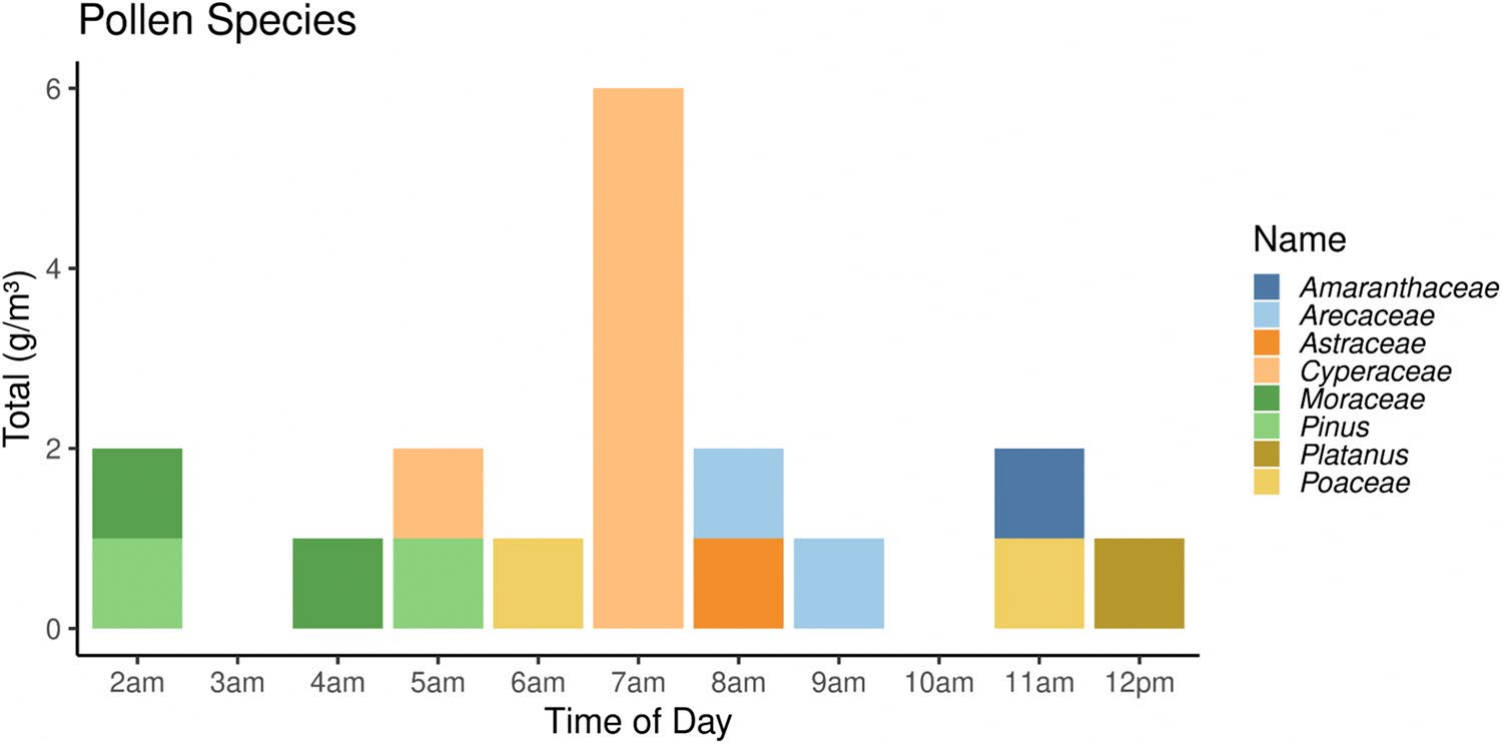
Pollen diurnally distribution pattern (2:00 am-12:00 pm). A maximum of Cyperaceae pollen (6 p.g/m^3^) was detected at 7:00 am

Diurnally, the airborne pollen and fungal spores collected during the Comrades Marathon showed a marked oscillation over the eleven-hour period. The graphic curves of fungal spores demonstrated a clear maximum concentration of 70 sp.g/m^3^ at 7:00 am (Fig. 9), while the maximum pollen concentration of 6 p.g/m^3^ was obtained at the same time (Fig. 10). The dominant fungal spores species showed that smuts peaks (39 sp.g/m^3^) were reached at 7:00 am, whereas *Cladosporium* (10 sp.g/m^3^) and *Alternaria* (5 sp.g/m^3^) spore peaks were recorded at 6:00 am.

### Botanical Survey

Our records are based on a field survey at the Drummond Halfway point on 29 August 2022. A total of twenty-four plant families and thirty-two species were identified in the sampled area. Relatively few of the taxa were flowering at the time of the survey. Those that were flowering, included trees such as *Cupressus* spp., and *Erythrina caffra* and herbaceous plants, predominantly *Chrysanthenmoides monilifera* and perennial succulent plants such as *Plectranthus neochilus* and *Crassula multicava.* There was no distinct pattern regarding the spatial distribution of taxa observed during the survey. Many of the identified taxa are animal-pollinated (e.g. *Erythrina caffra*) and produce low quantities of pollen, however other taxa, e.g. *Cupressus* spp., are wind-pollinated and known to produce large quantities of pollen which are well reflected in spore traps.

## Discussion

There are factors potentially at play that were not present at the race's beginning and end, which could explain the observed fluctuations in environmental conditions. Weather conditions on an event day are unpredictable, however as a general guideline, race organizers should provide runners and local authorities with forecasts of the event day and consider the deployment of weather stations at points along the route to monitor heat stress (and other weather conditions) in collaboration with medical responders, this could assist the direct deployment of personnel to sites where medical complications are likely. When examining the results it is clear a holistic perspective needs to be taken into account, as the conditions experienced on the route could be impacted by large scale circulation sudden changes in weather conditions as seen at Pinetown around noon. The microclimate conditions are particularly important as they are also seen at Pinetown where PM_2.5_ measurements compared with the nearby SAAQIS station differ significantly. The SAAQIS station, placed in a suburban area, measured maximum concentrations of 17 µg/m^3^ compared to the > 5000 µg/m^3^ at the station next to the route, exposed to direct smoke.

The fact that PM_2.5_ has instantaneous peaks along the route is a novel finding. Although there is no official data on spectator distribution, high PM concentrations at certain points could hint that the prevalence of PM might be higher at popular spectator spots. The public often lines up along substantial sections of the route, engaging in festivities. A major consideration for us when reflecting on the PM_2.5_ data is the lack of standards for short term exposure, it is important to consider that our measurement represents a fraction of the time a runner is exposed to this PM level. If, however, the results are *extrapolated* (Venter et al. 2015) over the course of the event (and during their training and other events), runners' exposure might be significantly high. Because of the temporal nature of our measurements, the results cannot be compared to the World Health Organisation's (World Health Organization 2021) 24-h exposure limit of 10 µg/m^3^, and this requires further investigation. These short term exposures over the course of an event may or may not have an impact on respiratory health that is yet to be explored. This initial study might also be relevant for other sporting events, as PM_2.5_ levels could fluctuate throughout the event and the athlete’s training program.

There are other considerations that need to be taken into account with the PM_2.5_ results. Regional pollution sources can extend from far away, regional recirculation of atmospheric pollutants can impact local events, what we measure can come from far away. South Africa lies beneath a semi-permanent high-pressure cell, and PM can be very high due to the accumulation of PM under inversion (stable) conditions. In this case the large-scale circulation does not indicate this was evident but any event can be impacted due to regional circulation transporting pollutants from far away sources. PM_2.5_ is only a size fraction, next steps include source apportionment studies to examine what PM_2.5_ particles athletes are exposed to (Thangavel et al 2022).

According to our aerobiological survey at Drummond Halfway cut-off on the Comrades Marathon route, low counts of biological aerosols were observed, dominant allergenic fungal taxa, in order of frequency, included smuts, *Cladosporium*, *Alternaria*, *Nigrospora*, *Spegazzinia*, basidiospores, and *Pleospora*. In addition, fungal spore exposure can last for months as the fungal spore seasons last twice as long as the pollen seasons, leading to high patient susceptibility for a more significant proportion of the year (Hughes et al. 2022). Sensitisation varies according to the taxonomic genera and species of fungi, with *Alternaria* and *Cladosporium* recognised as important sensitisers (Idrose et al. 2020). The specific fungal spores types detected at Drummond have been observed to worsen symptoms, especially during their high concentration, in people diagnosed with asthma and allergic rhinitis (Denning et al. 2006).

Aeroallergens presented in the atmosphere during the Comrades Marathon have been known for their allergenicity and the provocation of respiratory allergy symptoms to sensitized patients. Among these, Cyperaceae (Sedges), *Cupressus* (Cypress), *Morus* (Mulberry), Poaceae (Grasses), Amaranthaceae (Pigweed), *Platanus* (Plane tree) and Combretaceae (Bushwillow) pollen types have been confirmed to cause significant allergic symptoms in susceptible individuals (D’Amato et al. 2017; Adeonipekun et al.^2018^). Daily pollen counts provide a snapshot of the atmosphere for a given area and typically represent the average daily concentration from a single sampler. A great deal of variability in the composition of regional pollen exists within the atmosphere because of vegetation land-cover patterns, fluctuations related to weather events and the distance between the emission source area and the sampler (Sanchez Goñi et al. 2022).

The diurnal variation obtained showed a peak between 7:00 and 8:00 am in the early morning. Several authors (Sadyś et al. 2015; Grinn-Gofroń et al. 2018; Qin and Li 2022) have noted the diurnal variation in airborne pollen and fungal spores. In a study conducted on the aerospora profile of Johannesburg, Ajikah et al. (2023) confirmed that meteorological parameters including temperatures, relative humidity, wind speed and precipitation, in that order of importance, significantly influenced the composition of the atmospheric aeropsora content in both hourly and daily periods of time. This information is of particular importance and may help inform preventive management strategies for allergic-sensitized athletes hoping for peak performance at a specific hour during the race.

Planning future studies involving micro-climate measurements at sporting events calls for crucial ethical and logistical considerations. First, obtaining appropriate permissions and consent from race organizers and governing bodies is essential and ensuring the research process and its equipment do not impact the athlete’s or the event's organization. Researchers should remain neutral observers, respecting the athletes' participation in the event.

From a logistical perspective, research instruments should be easily relocatable and safety measures considered for high-crime hotspots. Power supply should be reliant on long-lasting battery setups to ensure uninterrupted operation. The complexity of the terrain should inform the number of monitoring stations for an accurate representation of the microenvironment. These stations need to be well-equipped with essential instruments, with diligent attention to calibration. Strategic placement of instruments, near timing mats or through the use of athletes' online tracking data, can effectively monitor athlete progress. Real-time sampling of bioaerosols provides valuable information on circulating aeroallergens before and during the sports event. By carefully addressing these considerations, more effective and efficient research studies can be performed.

## Conclusion

The 2022 Comrades Marathon was the first event in South Africa where weather stations and particulate monitoring instrumentation were deployed. On the day risk related to heat stress was low in the afternoon, but still important for the organizers to consider targeted intervention methods as slower runners (exposed to longer durations) might be more at risk. The study provided data on PM_2.5_ concentrations. The majority of PM_2.5_ readings from the 5 of the 7 stations were within normal ranges, however there were instances of higher values at two stations at specific times of the day. These instantaneous values highlight the need for research on athletes training exposure levels and if there is an effect on athlete health and performance. Currently there is no standard regarding short term PM_2.5_ exposure (such as a runner passing an instantaneous high PM source) and therefore there is a need for more research into the effect of pollutant inhalation during high levels of physical activity. Diurnal variations in the concentrations of fungal spores and pollens were observed, peaking at 7:00 am for both. The low count of aerospora present in the air during the race day might have no significant impact on an athlete's respiratory health.

As climate change may lead to an increased frequency of heat waves, extreme heat days and the release of aerospora, understanding and monitoring the effects of adverse environmental conditions is becoming increasingly important, and effective forecasting, pre-screening, and race day monitoring can help minimize these impacts. The present study during the Comrades Marathon highlights the importance of micro-climates. This information may be of importance to assist organizers to enhance safety for future events. Finally this study provides data for future weather and environmental monitoring at mass participation sporting events.

## Supplementary Information

Below is the link to the electronic supplementary material.

Supplementary file1 (DOCX 2020 KB)

## Data Availability

Data is available on request to the corresponding author and on agreement by all partner institutions.
