# Supplementary material for: Healthy environments for athleTes (HEAT): environmental conditions along a 90 km ultra-marathon event, South Africa
Source: Int J Biometeorol. 2024 Jun 13;68(9):1757–71. doi: 10.1007/s00484-024-02703-8 (PMC11461593; doi:10.1007/s00484-024-02703-8)
Supplement: Supplementary file 1 — Supplementary file1 (DOCX 2020 KB) [file 484_2024_2703_MOESM1_ESM.docx]

Suggestion for Supplementary Information


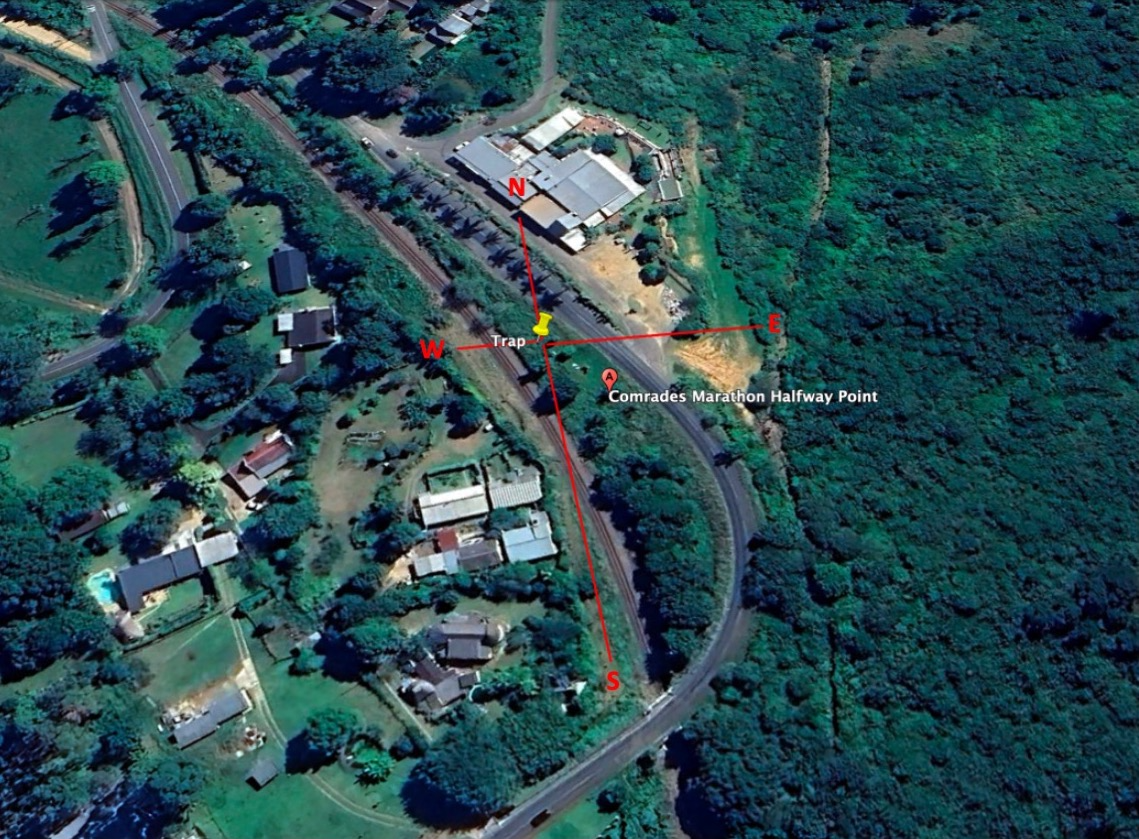


Figure S1. Google Earth image outlining the design of the vegetation survey used at the Comrades Halfway point at Drummond. Transect lines radiating outwards from the central spore trap location are indicated in red and follow the compass points. The south transect (S) extended the full 100 m, however the remaining three transects were truncated due to property boundaries curtailing access.
